# Sonochemical Effects on 14 Flavonoids Common in Citrus: Relation to Stability

**DOI:** 10.1371/journal.pone.0087766

**Published:** 2014-02-06

**Authors:** Liping Qiao, Yujing Sun, Rongrong Chen, Yu Fu, Wenjuan Zhang, Xin Li, Jianchu Chen, Yan Shen, Xingqian Ye

**Affiliations:** 1 College of Biosystems Engineering and Food Science; Zhejiang Key Laboratory for Agro-Food Processing; Fuli Institute of Food Science; Zhejiang R & D Center for Food Technology and Equipment;Zhejiang University, Hangzhou, PR China; 2 Department of Food Science and Technology, Zhejiang University of Technology, Hangzhou, PR China; University of Central Florida, United States of America

## Abstract

The sonochemical effects of ultrasound (US) treatment on 14 flavonoids representing the main flavonoids in citrus fruit were investigated in a standard mixture by stability evaluation of a model system. Degradation products were further tentatively identified by Fourier transform infrared spectroscopy and high-performance liquid chromatography–ultraviolet detection–electrospray ionization tandem mass spectrometry. Thirteen flavonoids (i.e., eriocitrin, narirutin, neohesperidin, quercitrin, eridictyol, didymin, naringenin, luteolin, sinensetin, nobiletin, tangeretin, naringin, and hesperidin) were fairly stable whereas quercetin was degraded significantly by US treatment. The types of solvent and temperature used were important factors that determined the resulting degradation reactions. The degradation rate of quercetin was highest in 80% ethanol aqueous solution and decreased with increasing temperature. Longer US durations caused increases in the extent of quercetin degradation. Liquid height, ultrasonic intensity, pulse length, and duty cycle of US affected degradation rates but did not change the nature of degradation of the flavonoids. Four types of reactions occurred simultaneously for quercetin under US treatment: oxidation, addition, polymerization, and decomposition. Eight degradation products were tentatively identified as dimer, alcohol addition, oxidation, and decomposition products.

## Introduction

Flavonoids are a class of naturally occurring compounds widely distributed in plants with a C6-C3-C6 carbon framework. Over 6,000 compounds have been identified from various combinations of multiple hydroxyl, methoxyl, *C*-glycosylation, and *O*-glycoside group substituents on the basic benzo-γ-pyrone [1]. Citrus fruits play an important role in the human diet because of their health benefits and high flavonoids and nutrient contents. Flavanone glycosides, namely, narirutin, naringin, hesperidin, and neohesperidin, are the major flavonoids in citrus fruits [2]. Recent studies have found that the biological properties of flavonoids include antioxidant, anti-inflammatory, and antimicrobial activities, as well as several other activities associated with atherosclerosis and cancer prevention [3], [4], [5].

Flavonoids are sensitive to degradation because of their hydroxyl and ketone groups and unsaturated double bonds. Previous studies have reported the effects of different processing treatments on the stability of flavonoids [6], [7]. For example, roasting buckwheat results in a 80% loss of rutin at 160°C for 30 min. Boiling, frying, baking, sautéing, steaming, and microwaving also result in different degrees of flavonoids reduction similar to those observed under mechanical processes (e.g., chopping and pressing).

Ultrasound-assisted extraction has been widely used for bioactive compounds extraction because of its accessibility, high extraction rate, and efficiency [8]. However, ultrasound (US) may exert sonochemical effects on extracts and some chemical reactions may be triggered or accelerated. For example, US treatment causes the degradation of carotenoids [9] and phenolic acids [10], as well as the aggregation and decomposition of polysaccharides [11]. Several papers have reported that sonication degrades or oxidizes flavonoids. Paniwnyk et al. [12] found that aqueous US methods result in lower rutin yields than conventional extraction when the US duration is longer than 20 min. Robak et al. [13] reported the US-induced oxidation of flavonoids in 1991. Despite the number of available studies on US methods, however, a few recent studies have considered whether or not ultrasound as a means of extraction affects the stability of the flavonoids. Only Biesag has examined the stability of selected flavonoids in honey [14] and maize [15] during sonication.

Whether deleterious effects or unwanted reactions on earth would occur during the process of extraction flavonoids from citrus by ultrasound treatment? It is necessary to verify this problem. However, the complicated sample system makes it difficult to determine and analyze the possible chemical reactions of target compounds in realistic conditions. The nature of chemical reactions would be generally identical in model system and realistic conditions and only some difference in their decomposition rates [15]. Therefore, a model system was developed to investigate this problem. The aim of this study was the determination of common US treatment factors (solvent, temperature, ultrasound intensity, liquid height, pulse length and duty cycle) on the stability of the major flavonoids commonly found in citrus in a model system, and the corresponding degradation products were analyzed. The results may be expected to briefly reflect the possible changes of flavonoids under ultrasound treatment and provide some theoretical basis for the ultrasound practical application to extract flavonoids from citrus.

**Table 1:** Effect of solvent on the stability of 14 standing (CK) and ultrasound (US)-treated flavonoids.

Solvent	Methanol	Ethanol	80% methanol	80% ethanol	Water
Eriocitrin (CK)	8.11±0.15	8.03±0.09	7.96±0.14	8.13±0.10	8.15±0.17
(US)	8.07±0.18	7.89±0.28	7.80±0.26	7.81±0.25	8.04±0.26
Narirutin (CK)	8.09±0.17	8.12±0.10	8.01±0.09	8.10±0.14	8.14±0.08
(US)	8.04±0.11	8.06±0.23	7.93±0.22	8.02±0.15	8.07±0.19
Naringin (CK)	8.07±0.12	8.03±0.16	8.00±0.15	8.12±0.09	8.16±0.16
(US)	8.01±0.20	7.95±0.26	8.02±0.21	8.06±0.14	8.13±0.17
Hesperidin (CK)	8.13±0.17	8.10±0.25	7.94±0.18	8.07±0.10	8.17±0.09
(US)	8.04±0.23	8.00±0.16	7.87±0.19	7.98±0.25	8.08±0.26
Neohesperidin (CK)	8.09±0.16	8.12±0.10	7.99±0.07	8.10±0.11	8.12±0.08
(US)	8.01±0.15	8.05±0.18	7.91±0.14	8.00±0.24	8.05±0.16
Quercitrin (CK)	8.00±0.18	8.10±0.13	7.98±0.20	8.06±0.19	8.18±0.26
(US)	7.89±0.24	7.91±0.20	7.75±0.21	7.91±0.26	8.04±0.17
Eridictyol (CK)	8.08±0.09	8.02±0.13	7.95±0.17	8.05±0.20	8.14±0.14
(US)	7.86±0.28	7.90±0.19	7.64±0.23	7.30±0.24	8.05±0.20
Didymin (CK)	7.96±0.10	8.06±0.18	7.97±0.11	8.11±0.17	8.13±0.11
(US)	7.81±0.24	7.95±0.20	7.84±0.18	8.02±0.28	8.04±0.21
Quercetin (CK)	8.08±0.17	8.07±0.24	8.08±0.24	8.12±0.16	8.10±0.17
(US)	6.46±0.21	6.81±0.13	7.11±0.15	3.54±0.30	6.39±0.19
Naringenin (CK)	8.03±0.18	8.12±0.13	8.01±0.10	8.08±0.16	8.18±0.17
(US)	8.00±0.16	8.05±0.26	7.87±0.19	7.97±0.21	8.09±0.19
Luteolin (CK)	8.05±0.17	8.09±0.12	8.02±0.16	8.10±0.18	8.14±0.14
(US)	7.94±0.22	8.00±0.20	7.88±0.19	7.65±0.18	8.02±0.15
Sinensetin (CK)	8.07±0.11	8.05±0.15	8.03±0.14	8.09±0.16	8.17±0.17
(US)	8.04±0.26	8.03±0.31	7.92±0.18	7.99±0.29	8.14±0.23
Nobiletin (CK)	8.09±0.14	8.07±0.17	8.02±0.10	8.10±0.12	8.13±0.19
(US)	8.01±0.27	7.98±0.31	7.85±0.22	8.04±0.28	8.05±0.15
Tangeretin (CK)	8.13±0.11	8.08±0.17	8.07±0.13	8.16±0.10	8.17±0.16
(US)	8.02±0.18	7.98±0.25	7.95±0.19	8.05±0.27	8.03±0.20

## Materials and Methods

### Chemicals

HPLC-grade methanol and formic acid were purchased from Tedia Company, Inc. (Fairfleld, OH, USA). Ethanol and methanol (analytical grade) were purchased from Sinopharm Chemical Reagent Co. (Shanghai, China). Water was purchased from Hangzhou Wahaha Group Co., Ltd. (Hangzhou, China). Eriocitrin, narirutin, neohesperidin, quercitrin, eridictyol, didymin, quercetin, naringenin, luteolin, sinensetin, nobiletin, and tangeretin were purchased from Sigma (St. Louis, MO, USA). Naringin and hesperidin were obtained from the National Institute for the Control of Pharmaceutical and Biological Products (Beijing, China). The purity of all of the standards was beyond 95%.

### Ultrasound Treatment

In this study, six factors have been investigated, including solvent (methanol, 80% methanol, ethanol, 80% ethanol, water), temperature (−5, 5, 25, 45, 65°C), ultrasound intensity (10.19, 15.29, 15.80, 16.31, 20.89, 23.95 W/cm^2^), liquid height (2, 4, 6, 8, 10, 12 cm), pulse length (0.2, 0.5, 1, 2, 4, 8 s) and duty cycle (33.3%, 40%, 50%, 66.7%, 100%). US treatments were conducted with a probe ultrasonic processor (JY92–IIDN, Ningbo Scientz Biotechnology Co., Ningbo, China) with the following parameters: maximum US power output, 900 W; frequency, 20–25 kHz; and horn microtip diameter, 10 mm. Standard flavonoids solutions of 8.0 µg/mL were mixed in same volumetric flasks and 30 ml of mixtures were kept in brown glass tubes (3 cm in diameter; 10–20 cm in height). The tubes were immersed in a low-temperature water bath (DC–1006, Safe Corporation, Ningbo, China) to maintain a constant temperature. Five solvents were chosen not only because they are frequently reported for extraction flavonoids, but also they may cause different sonochemical effect. Additionally, we want to know whether flavonoids stability would be affected when ultrasound intensity or temperature changes. The solutions were then treated by US with the following parameters (except for the tested factor): probe position, 1 cm from the top of the extraction cell; liquid height (the distance from the horn microtip to the bottom of the tube), 4 cm; temperature, 5°C; pulse mode: on, 2 s and off, 2 s; treatment time, 20 min; and US intensity, 15.29 W/cm^2^. Samples obtained by standing under the same conditions were used as controls (CK). The ultrasound treated samples (US) and CK solutions were filtered through 0.45 µm polyvinylidene fluoride microfiltration membranes (Shanghai Xingya Purification Material Co., Shanghai, China) and subsequently stored at –18°C for further HPLC analysis.

### Calculation of US Intensity

The ultrasonic intensity emitted from the probe microtip was calculated using the following formulas [16]:
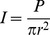
(1)

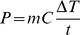
(2)where *I* is the US intensity (Wcm^−2^), *P* is the ultrasonic power delivered to the medium (W), r is the radius of the probe microtip (cm), m is the mass of the solvent (Kg), C is the specific heat capacity (J Kg^−1^K^−1^), T is the temperature (K), and t is the sonication time (s).

The actual ultrasonic power dissipated into the medium is lower than the nominal output power because of losses that occur in the US system. Thus, we measured the actual US intensities at six different levels by the calorimetric method.

A certain duration (180 s) was selected, and the levels of US power output were adjusted to 5%, 15%, 25%, 35%, 45%, and 65% of the total US power output (900 W) to obtain corresponding US intensities of 10.19, 15.29, 15.80, 16.31, 20.89, and 23.95 W/cm^2^, respectively.

### Analytical Method for Flavonoids

Ultra-fast liquid chromatography (UFLC) analysis of flavonoids was conducted on an LC–20 system (Shimadzu) linked to an SPD–M20A instrument. A 10 µL aliquot of the sample solution was injected into a reversed phase 250 mm×4.6 mm, ID 5 µm ZORBAX SB-C18 column (Agilent Technologies, USA). The mobile phase was composed of (A) 0.1% aqueous formic acid and (B) methanol. Gradient elution was performed as follows: 0–20 min, 37%–50% B; 20–35 min, 50%–80% B; and 35–40 min, 80%–100% B. The column thermostat was set to 25°C and the flow rate was 0.7 mL/min, similar to the conditions specified in our previous report [17]. The detection wavelength was set to 283 nm for flavanones, 330 nm for flavones, and 367 nm for flavonols.

All flavonoids were quantified with external standards by UFLC analysis. The concentrations of flavonoids were expressed as micrograms per milliliter solution volume (µg/mL). Fourteen different standard stock solutions with varying concentrations were prepared. The linear regression equation obtained within the range of 2.0 µg/mL to 20 µg/mL showed an R^2^≥0.996 for all of the measured flavonoids. The repeatability of intraday analysis showed a relative standard deviation (RSD) of≤3% (n = 3). Among the flavonoids, hesperidin showed the maximum limit of detection at 0.04 µg/mL, and narirutin showed the minimum limit of quantitation at 0.05 µg/mL.

### Determination of Degradation Products by FTIR Spectroscopy

The functional groups of degradation products obtained at 5–25°C were analyzed by FTIR spectroscopy. FTIR spectra of the degradation products were obtained by a Nicolet Avatar370 instrument (Thermo Fisher Scientific, USA) at wavenumbers ranging from 400 cm^−1^ to 4000 cm^−1^. The spectral resolution was 1 cm^−1^ and the collection time was approximately 1 min. Peaks analysis of the FTIR spectra obtained was performed using OriginLab 7.5.

### Determination of Degradation Products by HPLC-UV-ESI-MS/MS

High-performance liquid chromatography–ultraviolet detection–electrospray ionization tandem mass spectrometry (HPLC–UV–ESI–MS) analysis was conducted on an Agilent–6460 Triple Quad LC–MS fitted with an ESI source. Aliquots of 10 µL were taken from the CK and US solutions and injected directly into a 100×2.0 mm CAPCELL PAK C18 MG S3 column with a particle size of 2.9 µm (Shiseido Co., Japan). Elution was conducted in gradient mode using two components: A, 0.1% formic acid in water; and B, 0.1% formic acid in acetonitrile. For quercetin analysis, the gradient was 5%–65% B for 60 min. The column thermostat was set to 40°C and the flow rate was 0.2 mL/min. Data acquisition and processing were performed using Mass Hunter software. Negative ion mass spectra of the column eluates were recorded in the range of *m/z* 50–1000. Different collision energies (5, 15, 25, and 35V) were set to determine the representative product ions of peaks 1 to 8 in ESI–MS/MS mode. Other parameters were set as follows: fragmentor, 135 V; gas temperature, 325°C; and gas flow, 5 L/min. The sheath gas temperature was 350°C, and the sheath gas flow was 11 L/min. The capillary voltage was set to 3500 V for the negative ion mode.

### Statistical Analysis

Each treatment was replicated three times and mean values were reported expresing as means ± SD. All of the data were subjected to statistical analyses using SPSS 16.0 (SPSS Inc., Chicago, Illinois, USA). The effect of each US factor on the stability of flavonoids was tested by one-way ANOVA and Duncan’s multiple range tests. Mean differences at *p*<0.05 were considered significant.

## Results and Discussion

### Effect of Solvent on the Stability of the Flavonoids

**Figure 1:**
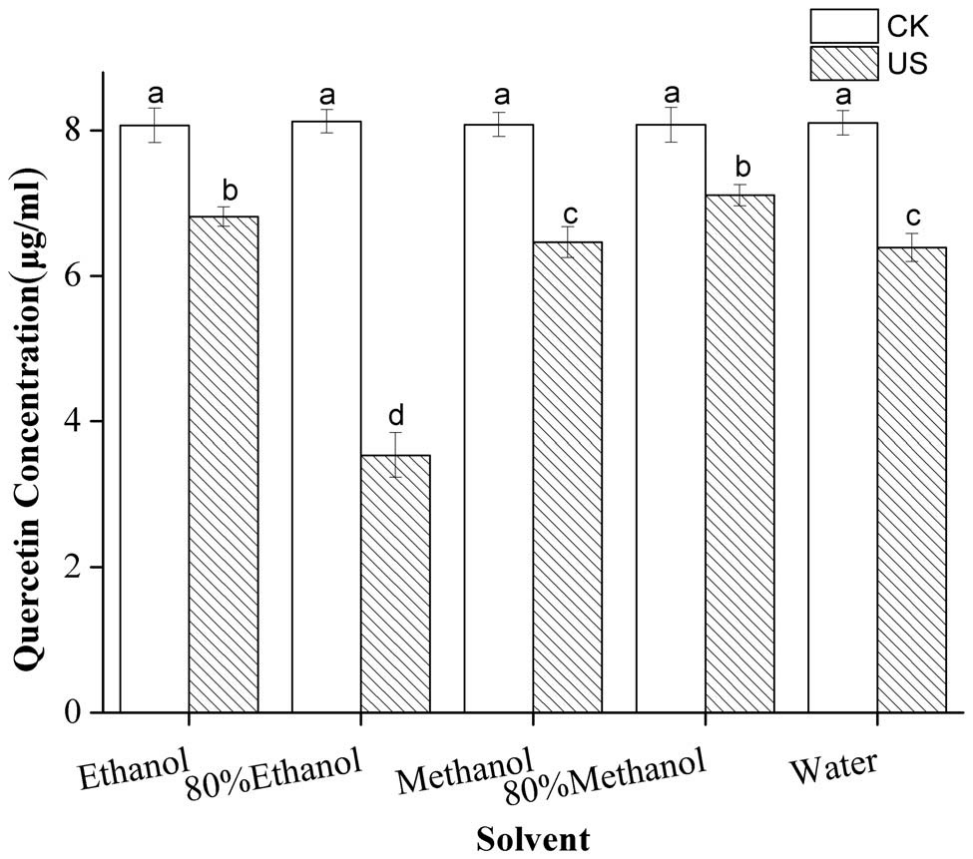
Effect of solvent on the stability of quercetin under ultrasound treatment (US) and standing (CK). Different letters on the bars show significant difference (*p*<0.05) according to Duncan’s multiple range tests.

Flavonoid standards representing the flavonoids in citrus fruits were mixed with different extraction solvents under US. The effect of different solvents at 5°C, with probe depth/liquid height 1 cm/4 cm, on/off 2/2 s, US intensity 15.29 W/cm^2^ under sonication and standing for 20 min on the stability of the 14 flavonoids is presented in Table 1. Among the flavonoids, only quercetin was degraded significantly (*p*<0.05); the rest appeared fairly stable in the solvents. The quercetin degradation rates obtained from the five solvents were markedly different (Fig. 1). US treatment caused strong chemical effects on quercetin in 80% ethanol and yielded the lowest concentration of quercetin among all of the solvents examined. Compared with untreated samples, the concentration of quercetin in 80% ethanol under US decreased by 56%. The highest concentration of quercetin was found in 80% methanol, in which only 12% quercetin was degraded under US treatment.

The stability of the flavonoid standards in a model system depends on the nature of the flavonoids and the extraction solvent used for US. Sonochemical effects may vary in different solvents. Paniwnyk [12] found that US in aqueous solvent produces large amounts of highly reactive hydroxyl radicals that interact with extracts and induce rutin degradation; however this reaction is not found when pure methanol is used with sonication for 60 mins. Flavonoids possess different group substituents and chemical structures that affect their stability. As indicated in a previous publication, the instability of the 3-hydroxyl group in flavonoids results in oxidation of flavonol aglycones and their 7-glycosides; by contrast, flavonol 3-glycosides, flavones, and flavanones remain stable during sonication [13]. A similar result was observed by Biesaga et al. [14], who reported that higher numbers of hydroxyl groups promote the degradation of flavonoids whereas sugar moieties and methoxyl groups protect flavonoids from degradation under US conditions. Results of these analyses offer possible explanations for the present data, which indicate that quercetin is degraded to about half of its original concentration in 80% ethanol whereas the concentrations of other flavonoids, e.g., hesperidin and nobiletin, changes only slightly in the same solvent after US treatment.

Hesperidin is the most abundant flavanone glycoside in citrus. Citrus fruits also contain other important flavonoids, such as naringin, narirutin, and eriocitrin. The bioactivities of citrus polymethoxylated flavones, such as nobiletin and tangeretin have been frequently reported [2]. Thus, the stability of the 14 flavonoids was determined under different US conditions. A model system of the flavonoid standards was developed to simplify the study because the nature of chemical reactions in these two systems could be roughly uniform. The conditions tested were chosen according to the realistic conditions under which the flavonoids are generally extracted from citrus reported by our previous publications [18], [19] and other studies [15], [20], [21]. The five polar solvents (i.e., methanol, 80% methanol, ethanol, 80% ethanol, and water) used in the present study are common in flavonoids extraction. However, quercetin showed the lowest stability among 14 flavonoids during US in 80% ethanol in the present experiment. To investigate this special phenomenon, the following experiments were carried out in 80% ethanol.

### Effect of Temperature on the Stability of the Flavonoids

**Figure 2:**
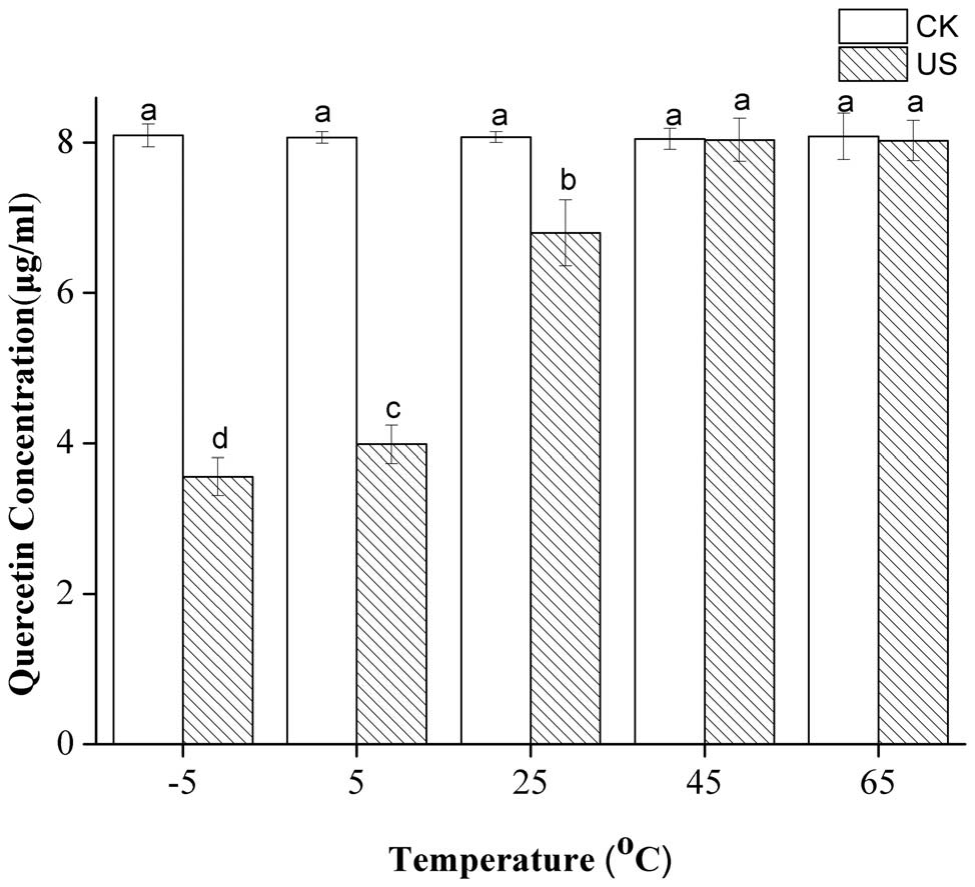
Effect of temperature on the stability of quercetin under US and CK. Different letters on the bars show significant difference (*p*<0.05) according to Duncan’s multiple range tests.

The 14 flavonoids were subjected to US in 80% ethanol at –5, 5, 25, 45, and 65°C. All of the flavonoid standards in 80% ethanol were completely dissolved at each temperature, and they were heat-stable in the tested temperatures [22]. Temperature appeared to be a significant factor (*p*<0.05) that influenced the flavonoids stability of quercetin in 80% ethanol (Fig. 2). Compared with untreated samples, the concentration of quercetin under US at –5°C, 5°C, 25°C, 45°C and 65°C decreased by 59%, 51%, 16%, 1% and 3% respectively. The 13 other flavonoids were not dramatically affected by temperature and remained stable under the following studied conditions (data not included). While a significant reduction in the amount of quercetin was observed as the extraction temperature decreased from 45°C to –5°C. No significant degradation of quercetin was found at 45 or 65°C.

Increasing temperature allows cavitation to be achieved at lower acoustic intensities [23] as a direct consequence of the increase in vapor pressure that occurs when the temperature of a liquid is increased. Among the different physical properties of solvents (e.g., surface tension, viscosity, and vapor pressure), vapor pressure shows the most significant effect (negative correlation) on cavitation intensity [24]. Thus, the cavitation intensity of US decreases with increasing temperature. The results obtained are in agreement with the results of a previous study, which indicated that using a lower temperature with US treatment results in higher degradation rates of caffeic acid standard [25] and oxidation of aqueous potassium iodide [26].

### Effect of US Intensity on the Stability of the Flavonoids

**Figure 3:**
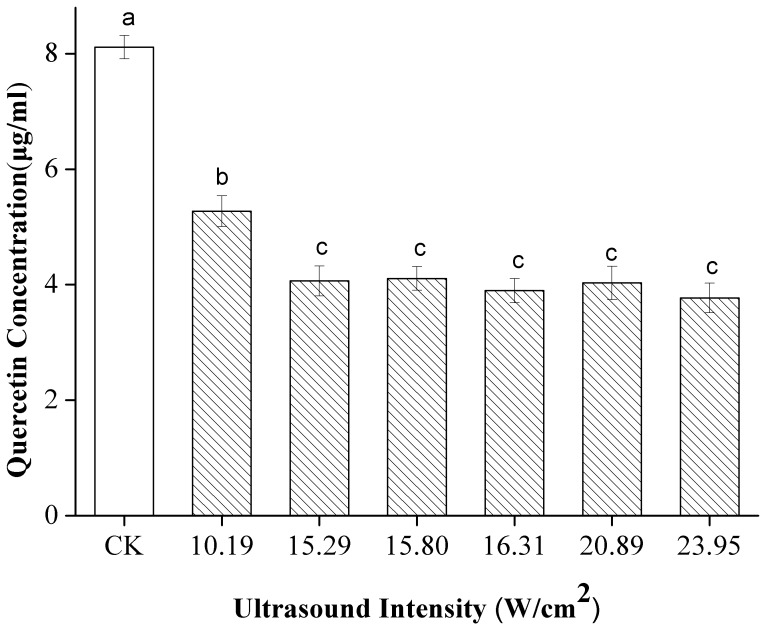
Effect of ultrasound intensity on the stability of quercetin under US and CK. Different letters on the bars show significant difference (*p*<0.05) according to Duncan’s multiple range tests.

Fig. 3 shows the effect of US intensity on the concentration of quercetin in 80% ethanol. US treatment at 10.19 W/cm^2^ yielded a high concentration (*p*<0.05) of quercetin; lower amounts of the flavonoids were obtained at higher intensities. No significant differences in quercetin concentration were observed at US intensities ranging from 15.29 W/cm^2^ to 23.95 W/cm^2^. While an increase in intensity generally results in an increase in sonochemical effects [23]. A possible explanation for this disagreement may lie in the range of US intensities applied. At low US intensities, cavitation bubbles are easily formed, and these bubbles collapse more energetically with increasing ultrasonic power [27]. Thus, increasing the US intensity helps promote sonochemical effects. When the power applied is relatively high, cavitation bubbles may grow extremely large and collapse weakly. The presence of excessive amounts of bubbles may also hamper the propagation of US waves and reduce cavitation effects [28]. Thus, quercetin concentrations are preserved better at 10.19 W/cm^2^ than at higher US intensities.

### Effect of Liquid Height on the Stability of the Flavonoids

**Figure 4:**
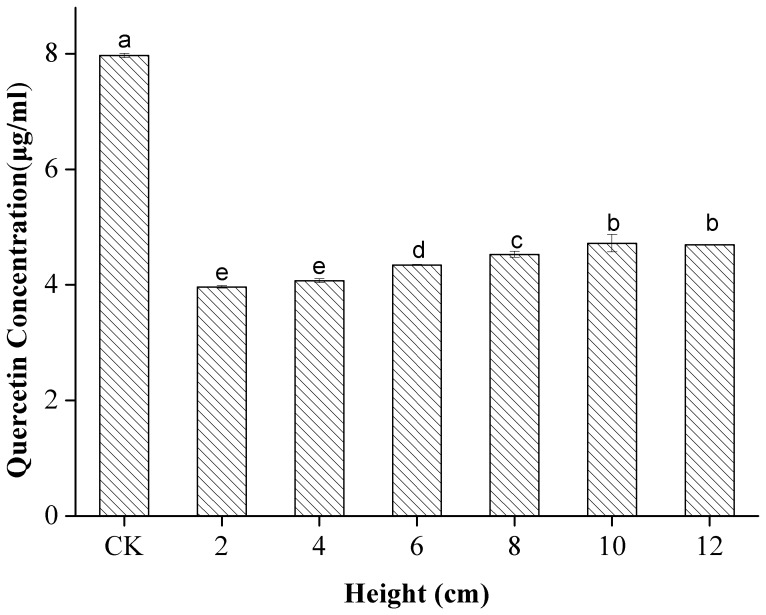
Effect of liquid height on the stability of quercetin under US and CK. Different letters on the bars show significant difference (*p*<0.05) according to Duncan’s multiple range tests.

The effect of liquid height (distance from the horn microtip to the tube bottom) on the quercetin concentration in 80% ethanol is shown in Fig. 4. Quercetin degradation decreased as the liquid height increased from 2 cm to 12 cm (*p*<0.05). For example, nearly half the available quercetin was degraded when the liquid height was 2 cm, whereas only three-eighths of the quercetin was degraded when the height was 12 cm. This result is attributed to the decrease in cavitation intensity with increasing height owing to the attenuation of the waves caused by absorption and scattering. Other researchers [29] have reported that the maximum power is present in the vicinity of the radiating surface of the US horn and that US intensities decrease rather abruptly as the distance from the radiating surface increases. Qiao [10] also observed small reductions in caffeic acid and sinapic acid as the liquid height increased during US treatment in 80% ethanol. However, in contrast to the findings of Qiao, another study [9] found that the concentration of β-carotene subjected to US in dichoromethane decreased markedly at heights ranging from 2 cm to 6 cm and then increased slightly at heights ranging from 6 cm to 12 cm. This disagreement observed between studies may stem from difference of the attenuation coefficients of the two solutions.

### Effect of Pulse Length on the Stability of the Flavonoids

**Figure 5:**
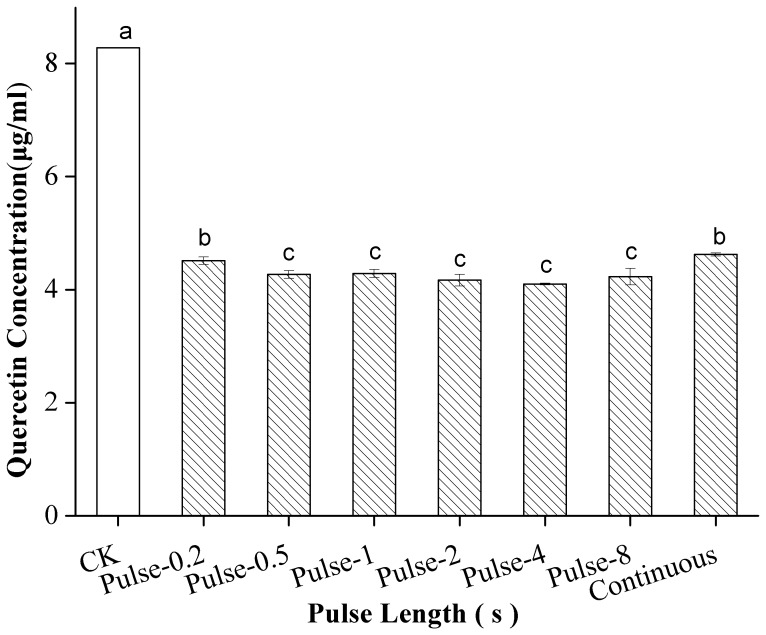
Effect of pulse length on the stability of quercetin under US and CK. Different letters on the bars show significant difference (*p*<0.05) according to Duncan’s multiple range tests.

The stability of the flavonoids in the standard mixture during US treatment was studied at different pulse lengths (all duty cycles fixed at 50%). Different pulse lengths caused the quercetin concentration to degrade significantly (*p*<0.05). Fig. 5 shows that the quercetin concentration obtained at pulses of 0.2 s was similar to that obtained in continuous mode. More serious degradation of quercetin was detected when the solution was subjected to US at pulses of 0.5, 1, 2, 4, and 8 s than when the solution was subjected to continuous US. Increasing in the pulse length from 0.5 s to 8 s in pulsed US mode did not result in significant differences in quercetin concentration.

In the present study, pulsed US showed different levels of efficacy with varying pulse lengths and pulse intervals; in other words, sonochemical activities were sometimes higher and sometimes similar to those observed under continuous US. These results differ from those found in Gutiérrez’s study [30], who reported that the efficacy of pulsed US was weaker than that of continuous US at higher intensities and similar to that of continuous US at lower intensities. However, according to Sun’s report [31], the sonochemical effects of pulsed US were milder than those of continuous US as the pulse length increased from 0.1 s to 1 s and more severe than that of continuous US as the pulse length increased from 1 s to 9 s (sample duty cycle fixed at 50%). Variations in the pulse durations and intervals applied may account for our observations. Pulses cannot produce chemical reactions immediately upon contact and instead require some time, denoted as the “activation time” τ_1_, to form gas bubbles of a suitable size. These bubbles disappear within a characteristic time, denoted as the “deactivation time” τ_2_. If the interval between the pulses T_off_>τ_2_, the following pulse has to reactivate the solution. However, if T_off_<τ_2_, the subsequent pulse can act on the bubbles formed by the preceding pulse and in this way be more chemically efficient. If the pulse length T_on_<τ_1_, no chemical effect occurs [31].

### Effect of Duty Cycle on the Stability of the Flavonoids

**Figure 6:**
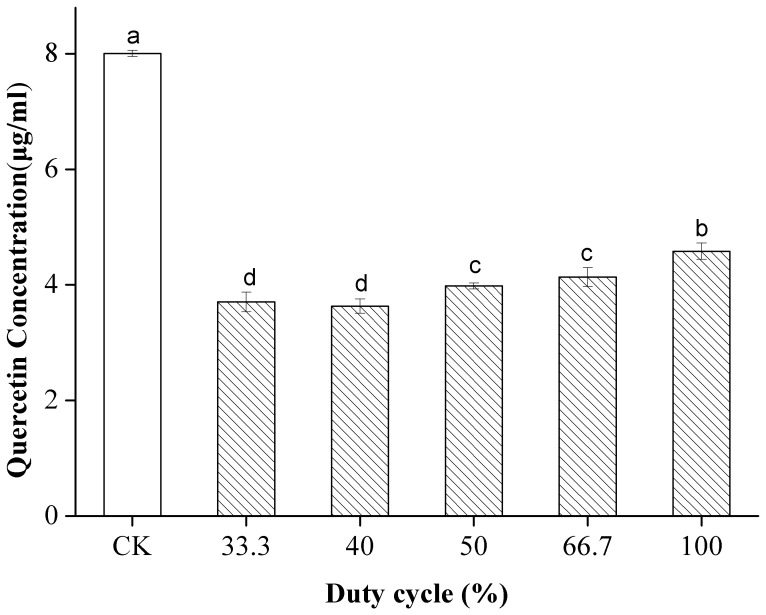
Effect of duty cycle on the stability of quercetin under US and CK. Different letters on the bars show significant difference (*p*<0.05) according to Duncan’s multiple range tests.

The effect of duty cycle on the concentration of quercetin in 80% ethanol was investigated at a pulse length of 2 s. The duty cycle denotes the percentage of pulse-on time relative to the sum of the pulse-off and pulse-on times. Fig. 6 shows that different duty cycles of US decreased the concentration of quercetin significantly and that quercetin was degraded less at higher duty cycles than at lower ones. The influence of duty cycle on the cavitation effects reported in different studies is inconsistent with that observed in the present work. Sun et al. [9] found that β-carotene shows a maximum degradation rate at a duty cycle of 66.7%. However, Luque–Garcí [32] reported that the duty cycle is not a significant factor for total fat extraction from oleaginous seeds. We believe that poor sonochemical effects result from higher duty cycles. At higher duty cycles, limited time is available for pulse intervals to release heat accumulated in the transducer under pulsed US. Thus, the weakest sonochemical effects may be obtained under continuous US because of the lack of time by which the heat accumulated in the transducer may be released [31].

### Time-course for Quercetin Degradation Under US Treatment

**Figure 7:**
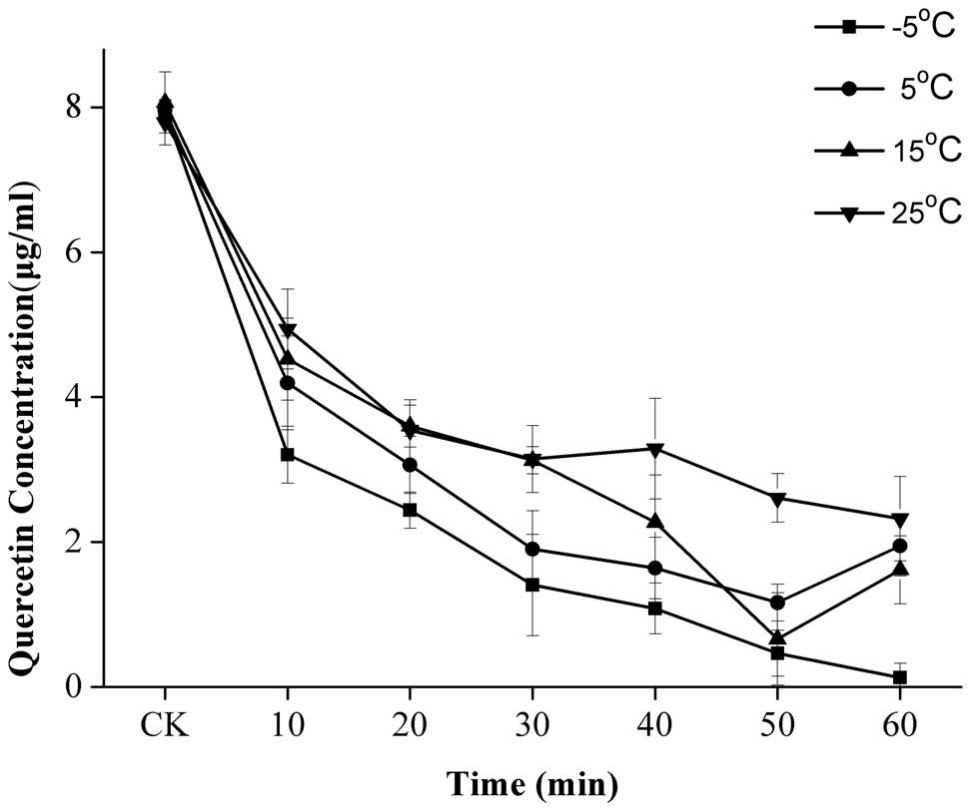
Time-course of quercetin degradation under US and CK at –5, 5, 15, and 25°C.

The time-course experiment on quercetin concentration (µg/mL) was conducted under US treatment at –5, 5, 15, and 25°C (Fig. 7) for 60 min based on the information shown in Fig. 2. Quercetin concentrations generally decreased over time during sonication. A sharp reduction in quercetin was observed within the first 10 min of US treatment, after which quercetin concentrations decreased slowly. These results may be explained by the fact that long treatment times may easily cause instrument fatigue and decrease US treatment effects.

### Degradation Product Analysis

**Figure 8:**
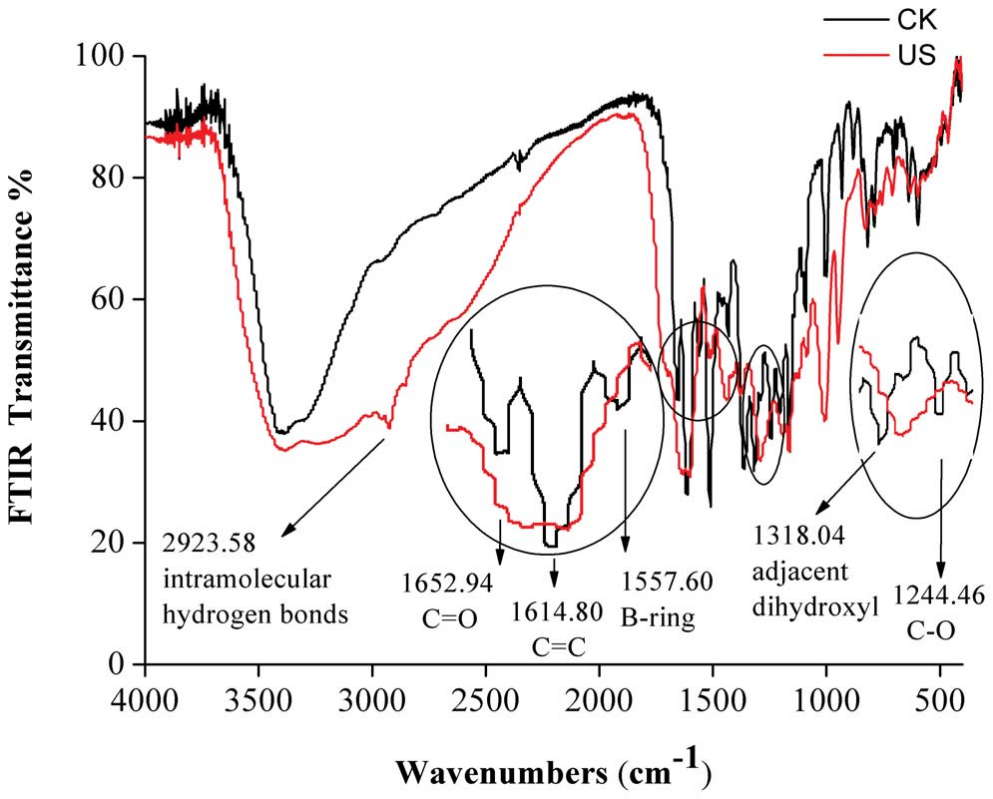
FT-IR spectra of US and CK treated quercetin obtained at different wavenumbers (cm^−1^) under at 5°C.

The FT-IR spectra of quercetin treated by US from –5°C to 25°C were similar; thus, we only present the degradation products of quercetin at 5°C as an example. Differences in the spectra of the CK and US samples may be observed (Fig. 8). A new peak at 2923.58 cm^−1^, corresponding to intramolecular hydrogen bonds, was observed after sonication in the US samples. Absorbance at 1652.94 (C = O stretching) and 1614.80 (C = C stretching) cm^−1^ disappeared, which suggests the reaction of ketone groups and unsaturated double bonds. B-ring may have changed because no corresponding signals could be observed at 1557.60 cm^−1^. The disappearance of the peak at 1318.04 cm^−1^ in the US spectra also indicated the reaction of adjacent dihydroxyl groups in B-ring after sonication [33]. From the vanishing of C-O vibration at 1244.46 cm^−1^, the fused heterocyclic C- ring of quercetin was suspected to be destroyed by US treatment.

**Figure 9:**
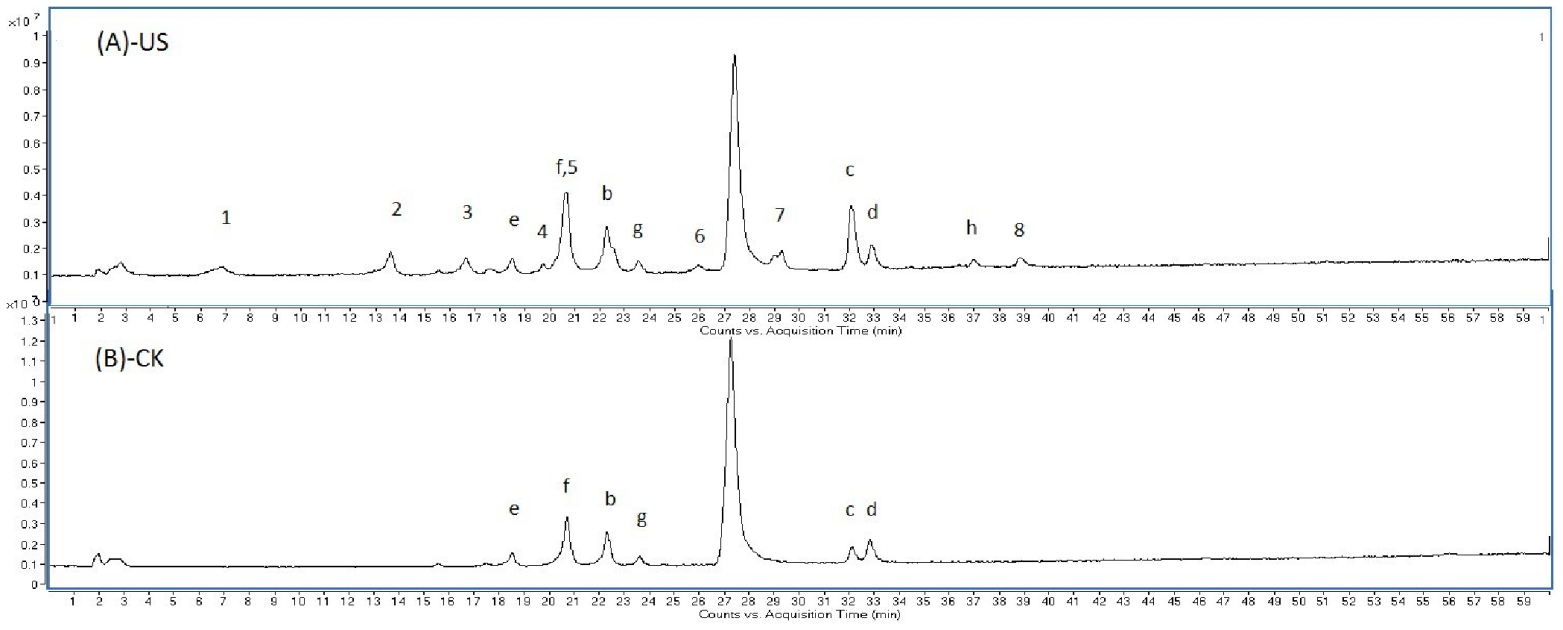
Negative-ion mode HPLC-ESI-MS total ion chromatograms of US and CK treated quercetin at 5°C.

**Figure 10:**
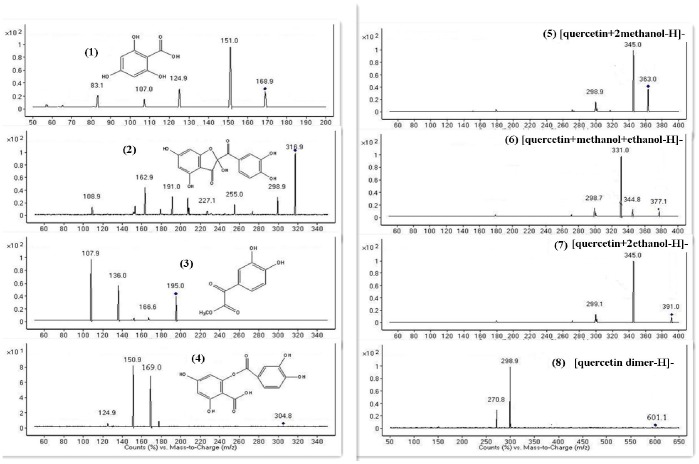
ESI-MS/MS spectra of HPLC peaks 1 to 8 obtained from quercetin treated by ultrasound and proposed structures of the corresponding products.

Several new chromatography peaks in the HPLC-UV spectra (254 nm) appeared after quercetin was subjected to US. To identify these peaks, HPLC-UV-ESI-MS (Fig. 9) and ESI-MS/MS were applied (Fig. 10). The corresponding ESI-MS and ESI-MS/MS spectral data of the degradation products of quercetin are shown in Table 2. All of the products have been reported previously, and some shared identical mass data information. Peak 1, eluted at 6.5 min, showed a quasi-molecular ion [M-H]^−^ at *m/z* 169.0 and four key product ions (151.0, 124.9, 107.0, and 83.1) from its representative ESI-MS/MS spectrum, which is consistent with the standard 2,4, 6-trihydroxybenzoic acid [35]. From the pseudo molecular ion [M-H]^−^ at *m/z* 316.9, the molecular weight of peak 2 was readily identified as 318; this peak is believed to be the oxidized product of quercetin, 2-(3,4-dihydroxybezoyl)-2,4,6-trihydroxy-3(2*H*)-benzofuranone. This benzofuranone derivative has been observed previously in the radical [34] and electrochemical oxidation of quercetin [36]. Peak 3 yielded a molecular ion [M-H]^−^ at *m/z* 195.0 and key product ions (136.0, 107.9). Comparison of these ions with [M+H]^+^ at *m/z* 197 obtained by CI-MS indicated that peak 3 may be methyl 3,4-dihydroxyphenylglyoxylate [34], [36]. Product 4 (Rt 19.5 min) was readily identified as 2-(3,4-dihydroxybezoyl)-4,6-dihydroxy-benzoic acid because it displayed a pseudo molecular ion and major fragment ions (304.8, 169.0) identical to those reported for synthetic standards in the literature [36], [37]. We confirmed that product 1 {2,4,6-trihydroxybenzoic acid ([M-H]^−^ at *m/z* 169.0)} was generated from product 4 on account of their similar ESI-MS/MS spectra and change in abundance (data not shown) with different collision energies. Peak 5 gave a quasi-molecular ion [M-H]^−^ at *m/z* 363.1 (363−301 = 2*31), which was reported previously as a dimethanol adduct of quercetin [34]. A key product ion at 345.0 was unexpectedly observed in the MS/MS spectrum. We confirmed that the molecular weight of peak 5 is 364 and believe it may be a dimethanol adduct of quercetin. Based on a pseudo molecular ion [M-H]^−^ at *m/z* 377.1 and two fragment ions at *m/z* 344.8 and 331.0, which demonstrate the loss of one methanol (377−345 = 32) and one ethanol (377−331 = 46), we identified product 6 as a methanol and ethanol adduct of quercetin [34]. Fig. 10 shows that product 7, eluted at 29.0 min, should be readily identified as a diethanol adduct of quercetin because it yielded a quasi-molecular ion [M-H]^−^ at *m/z* 391.0 (391−301 = 2*45) and a fragment ion at *m/z* 345.0 (345−301 = 44) by comparing with the data from the reference [34]. The last observed peak 8 showed a molecular ion [M-H]^−^ at *m/z* 601.1 and a key product ion at *m/z* 298.9. These ions indicate the formation of a dimer generated during the oxidation of quercetin. However, a dimer structure could not be identified among the possible structures of quercetin products previously reported [34], [38], [39], [40]. Several peaks were not identified in this work. For example, some peaks (peak a) could not be identified because of very low responses in the ESI-MS spectra. The other peaks (peak b - g) were not identified as they were impurities (<5%) proved by their presence in both the CK and US spectra (Fig. 9).

**Table 2:** Identification of new chromatographic peaks (HPLC-UV-254 nm) from quercetin treated by ultrasound.

Peak No.	HPLC Rt(min)	Molecular weight	HPLC-ESI -[MS]-MS^2^ (*m/z*)	Tentative identification	
1	6.5	170	[169.0]: 151.0, 124.9, 107.0	C_7_H_6_O_5_	2,4,6-trihydroxybenzoic acid
2	13.3	318	[316.9]: 298.9, 255.0, 162.9	C_15_H_10_O_8_	2-(3,4-dihydroxybezoyl)-2,4,6-trihydroxy-3(2*H*)-benzofuranone
3	16.4	196	[195.0]: 136.0, 107.9	C_9_H_8_O_5_	methyl 3,4-dihydroxyphenylglyoxylate
4	19.5	306	[304.8]: 169.0, 150.9, 124.9	C_14_H_10_O_8_	2-(3,4-dihydroxybezoyloxy)-4,6-dihydroxy-benzoic acid
5	20.4	364	[363.1]: 345.0, 298.9, 270.9	C_17_H_16_O_9_	dimethanol adduct of quercetin
6	25.7	378	[377.1]: 344.8, 331.1, 298.7	C_18_H_18_O_9_	methanol and ethanol adduct of quercetin
7	29.0	392	[391.0]: 345.0, 299.1	C_19_H_20_O_9_	diethanol adduct of quercetin
8	38.6	602	[601.1]: 298.9,270.8	C_30_H_18_O_14_	quercetin dimer

**Figure 11:**
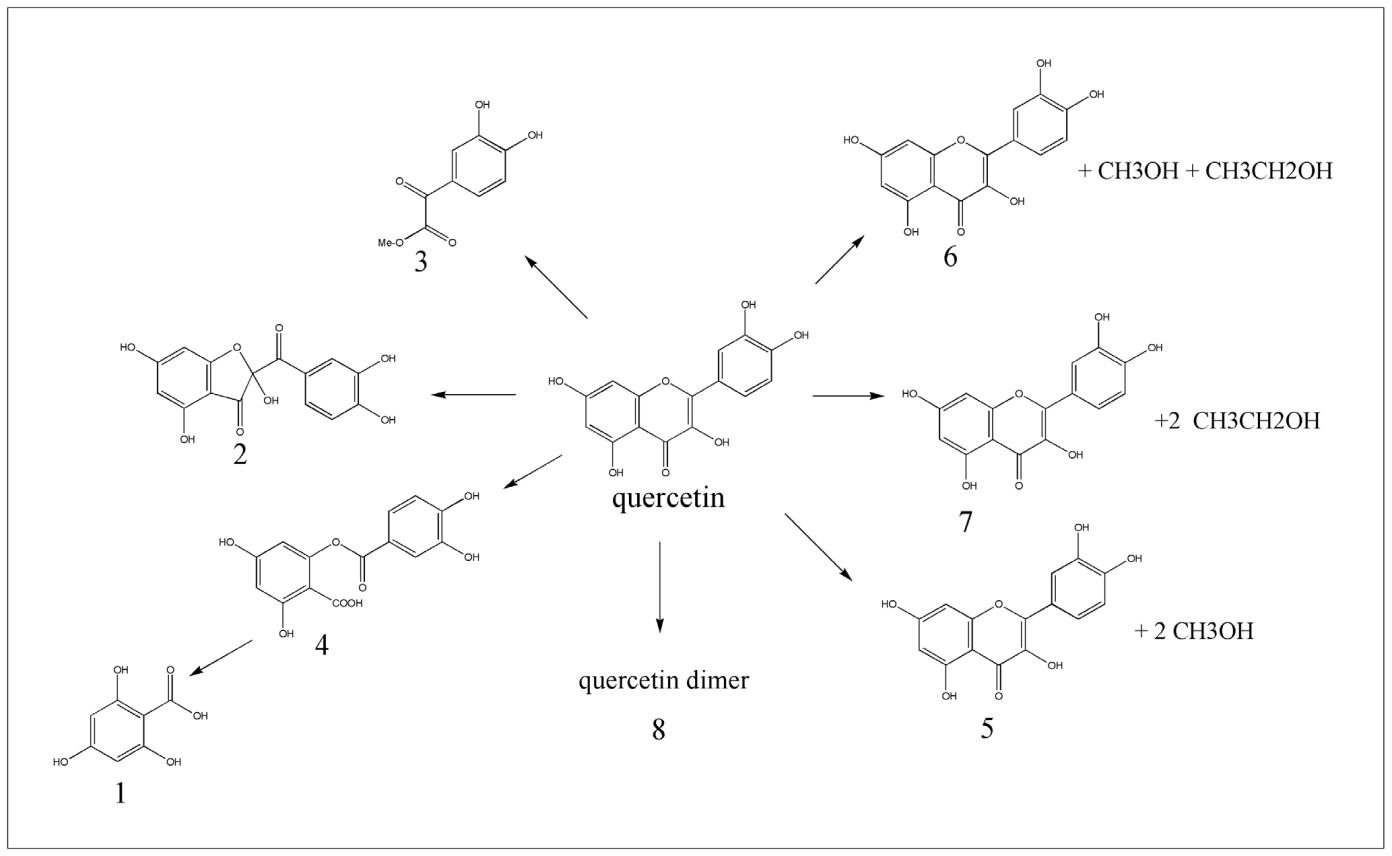
Proposed degradation mechanism of quercetin under ultrasound treatment.

From the ESI-MS, ESI-MS/MS, and FT-IR spectra of quercetin with and without US treatment, the oxidation, addition, polymerization, and decomposition reactions of quercetin may be concluded to occur simultaneously. The proposed degradation mechanism of quercetin under US is demonstrated in Fig. 11. The fused heterocyclic ring of quercetin serves as the reactive center for all products detected with an accompanying loss or gain of a substituent in the original C-ring unit during sonication [13], [34]. In the oxidation reaction, the pyranone C-ring of quercetin is converted to a furanone-carbonyl derivative (product 2). The first hypothesis on quercetin decomposition at a cleaved C-ring structure is based on the identification of products 1 and 3. The appearance of product 4 reinforces this idea C-ring group may also be the reaction site for polymerization and addition. In the present study, we have analyzed the molecular weights and functional groups of the degradation products of quercetin, basing our assignments on a comparison of mass spectra with literature reports. Independent characterization by other techniques, such as NMR spectroscopy, was difficult to accomplish because of the very low yields of some degradation products. Confirmation of the detailed structures of these products is recommended.

## Conclusions

To investigate the sonochemical effects on flavonoids common in citrus fruits under ultrasound treatment, we analyzed the stability of 14 flavonoids in a model system. In particular, we analyzed the effects of different US parameters on the degradation of the 14 flavonoids, examined the reaction times of the analytes, and identified the degradation products of quercetin. The results indicated that 13 of the 14 flavonoids (i.e., eriocitrin, narirutin, neohesperidin, quercitrin, eridictyol, didymin, naringenin, luteolin, sinensetin, nobiletin, tangeretin, naringin, and hesperidin) remained stable under US treatment and that only quercetin was degraded by the procedure. The type of solvent and temperature used were important factors in determining the degradation reaction of the flavonoids. Other factors, such as liquid height, US intensity, pulse length, and duty cycle affected the rate of degradation but not the nature of the reaction. Quercetin degradation rates decreased with increasing temperature. During US treatment of quercetin, oxidation, addition, polymerization, and decomposition reactions appeared to occur simultaneously. This study was hoped to provide some useful information on the application of the US technique for the extraction of flavonoids.
