# Correction: Sonochemical Effects on 14 Flavonoids Common in Citrus: Relation to Stability

**DOI:** 10.1371/journal.pone.0105647

**Published:** 2014-08-08

**Authors:** The PLOS ONE Staff

There is an error in the author contributions. The first two authors, Liping Qiao and Yujing Sun, should be noted as contributing equally to this work.
